# Epoxy-Modified Bismaleimide Structural Adhesive Film Toughened Synergistically with PEK-C and Core–Shell Polymers for Bonding CFRP

**DOI:** 10.3390/polym15061436

**Published:** 2023-03-14

**Authors:** Liwei Zhao, Xin Xu, Wanbao Xiao, Hongfeng Li, Hao Feng, Changwei Liu, Yingjie Qiao, Xuefeng Bai, Dezhi Wang, Chunyan Qu

**Affiliations:** 1 Institute of Petrochemistry, Heilongjiang Academy of Sciences, Harbin 150040, China; 2 College of Material Science and Chemical Engineering, Harbin Engineering University, Harbin 150001, China

**Keywords:** bismaleimide adhesive, synergistic toughening, composite joint, bonding performance, failure mode

## Abstract

Bismaleimide (BMI) resin-based structural adhesives have excellent heat resistance, with important applications demonstrated in the bonding of high-temperature BMI composites. In this paper, we report an epoxy-modified BMI structural adhesive with excellent properties for bonding BMI-based CFRP. We prepared the BMI adhesive using epoxy-modified BMI as the matrix and PEK-C and core–shell polymers as synergistic tougheners. We found that the epoxy resins improve the process and bonding properties of BMI resin but slightly reduce thermal stability. PEK-C and core–shell polymers synergistically improve the toughness and bonding performances of the modified BMI adhesive system and allow the maintenance of heat resistance. The optimized BMI adhesive exhibits excellent heat resistance, with a high glass transition temperature of 208.6 °C and a high thermal degradation temperature of 425.4 °C. Most importantly, the optimized BMI adhesive exhibits satisfactory intrinsic bonding and thermal stability. It has a high shear strength of 32.0 MPa at room temperature and up to 17.9 MPa at 200 °C. The BMI adhesive-bonded composite joint has a high shear strength of 38.6 and 17.3 MPa at room temperature and 200 °C, respectively, indicating effective bonding and excellent heat resistance.

## 1. Introduction

Carbon fiber-reinforced polymer (CFRP) is widely used in the aerospace field because of its many advantages over other materials, which include being lightweight and having high specific strength and fatigue and corrosion resistance [1,2,3], with benefits in terms of fuel efficiency, payload, service life, and aircraft safety. The development of joining technologies, including mechanical joining, welding, screw and rivet connection, and adhesive bonding, has driven the application of CFRP in engineering [4,5]. In the fabrication of CFRP structural components for aerospace applications, adhesive-bonding technology offers many advantages over other joining technologies [6,7]. The use of adhesive-bonding technology can reduce the weight of structural components, improve manufacturing efficiency, reduce manufacturing costs, and avoid stress concentration caused by drilling and structural damage to CFRP [8]. Therefore, bonded composite structural components are widely used in aircraft [9]. Research on composite bonding mainly involves the design and performances of adhesives [10,11,12], the mechanical behavior and properties of adhesive-bonded composite joints [13,14,15], analysis methods [4,16], and numerical simulations [17,18,19]. The methods for bonding composite materials mainly include co-curing, co-bonding, and secondary bonding. The factors affecting the bonding performance of CFRP joints are mainly adhesive [20] and CFRP properties [21], and environmental factors [22,23]. Behera et al. [24] used fiber-reinforced epoxy adhesive as a binding agent to prepare composite-bonded single-lap joints with improved tensile strength and durability. Luo et al. [25] prepared graphene-modified high-temperature-resistant adhesives for bonding carbon/carbon composites. The bonding strength was 63.09% higher for the specimen with 1.5 wt% of graphene than those with 0.0 wt%. Peiris et al. [26] studied the mechanical behavior of double-lap tensile shear joints of CFRP strips with different moduli and found that CFRP strips with high modulus exhibited higher bonding strength. In addition, the influence of the composite material surface treatment on bonded joint performance is also important [27,28,29]. Surface treatment of the composite material was implemented by Bora [30] using a CO_2_ laser, which created micro-holes on the surface of the CFRP, thereby improving the mechanical interlocking effect on adhesive bonding and the bonding strength of the CFRP joints. Bello et al. [14] studied the fatigue crack growth in laser-treated, adhesively bonded composite joints, and their results show that uniform laser treatment increases the fatigue limit of the joints.

The rapid development of high-speed aircraft is dependent on high-performance CFRPs that are resistant to the high temperatures generated by frictional heat during high-speed flight [31]. Bismaleimide (BMI) resin-based CFRPs exhibit excellent heat resistance and mechanical properties under high temperatures [32,33], making them widely used in the field of large and high-speed aircraft. The adhesive used for bonding BMI CFRPs must have a good process and mechanical properties in addition to high-temperature resistance. In practice, the bonding of CFRPs requires such adhesives that contain the same or similar resin systems and curing systems. This is particularly important for co-curing processes. Due to the excellent heat resistance and humidity heat resistance, BMI structural adhesives are required for the manufacture of adhesive-bonded BMI CFRP structural parts rather than other types of adhesives. More importantly, the matrix of BMI adhesives and BMI composites are similar, resulting in a robust interface through covalent bonding during co-curing. However, most BMI resins with high heat resistance are generally brittle [34,35], making them unsuitable for the preparation of adhesives. Comparatively, more works have focused on improving the toughness of BMI resins [36,37] or composites [38], with fewer involving BMI structural adhesives and co-cured composite joints. BMI structural adhesives for CFRP bonding require in-depth research, as well as the adhesive-bonded composite joints. In this study, we designed a modification system and toughener system to prepare a BMI structural adhesive with excellent process performance, bonding performance, and toughness for bonding BMI-based CFRP. The BMI adhesive was prepared using epoxy-modified BMI as the matrix and PEK-C and core–shell polymers as synergistic tougheners. The curing agent in the epoxy resin system can react with BMI molecules to form covalent bonding, improving process performance and bonding performance and maintaining excellent heat resistance. The synergistic toughening system can form a good interaction with the matrix, which greatly improves the single-lap shear strength and peel strength of BMI adhesive. The prepared BMI structural adhesive was suitable for bonding BMI CFRPs and provided excellent thermal stability and mechanical properties. The optimized BMI adhesive exhibits excellent heat resistance, with a high glass transition temperature of 208.6 °C. Most importantly, the optimized BMI adhesive exhibits satisfactory intrinsic bonding stability, with a high shear strength of 32.0 MPa at room temperature and up to 17.9 MPa at 200 °C. The BMI adhesive-bonded composite joint exhibits a high shear strength of 38.6 and 17.3 MPa at room temperature and 200 °C, respectively, indicating effective bonding and remarkable heat resistance. We revealed that good interfaces are beneficial to the improvement of the mechanical properties of composite joints.

## 2. Experiments

### 2.1. Materials

**Figure 1:**
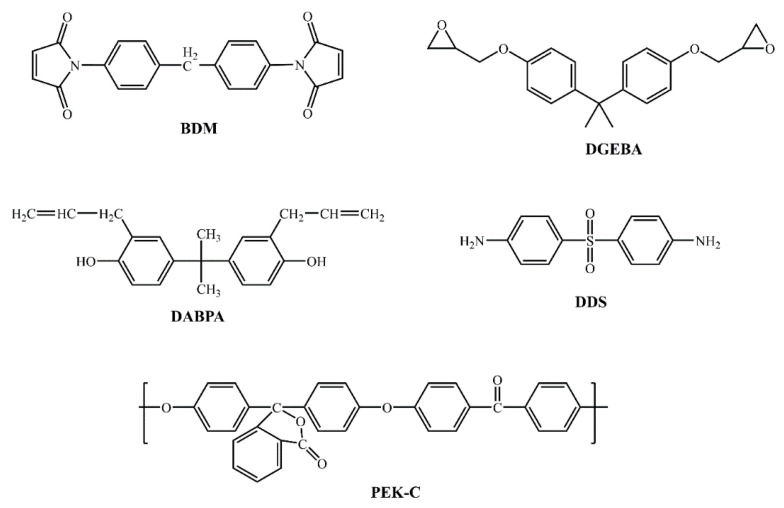
Chemical structures of adhesive components.

N, N-4,4′-diphenylmethyenebismaleimide (BMI) was purchased from Honghu Bismaleimide New Material Technology Co., Ltd. (Honghu, China). The 2,2′-diallylbisphenol A (DABPA), bisphenol A diglycidyl ether (DGEBA), and 4,4′-diaminodiphenyl sulfone (DDS) were purchased from Meryer (Shanghai) Chemical Technology Co., Ltd. (Shanghai, China), and polyether ketone (PEK-C) was provided by Changchun Institute of Applied Chemistry Chinese Academy of Sciences. Core–shell styrene-butadiene rubber was purchased from Kaneka Corporation (Tokyo, Japan). Aluminum alloy was purchased from Southwest Aluminum Industry (Group) Co., Ltd. (Chongqing, China). AVIC Composites Co., Ltd. (Beijing, China) provided BMI-based carbon fiber prepreg. The molecular structures of the chemicals are presented in Figure 1.

### 2.2. Preparation of BMI/DABPA Prepolymer and Epoxy-Modified BMI/DABPA Prepolymer

The molar ratio of BMI and DABPA was fixed at 1.0:0.87 for the preparation of the BMI/DABPA prepolymer. DABPA was added to a three-necked round bottom flask equipped with an agitator and heated to 135 °C, followed by the addition of BMI and stirring until a homogeneous and transparent liquid formed. The well-mixed BMI/DABPA prepolymer was obtained after being heated at 135 °C for 30 min. To prepare the epoxy-modified BMI/DABPA resin, DGEBA, and DDS were mixed at 120 °C at a stoichiometric ratio of 2:1 until a transparent homogeneous mixture formed. The content of the DGEBA/DDS mixture was determined by the weight of the BMI/DABPA prepolymer, as shown in Table 1. The DGEBA/DDS mixture and the BMI/DABPA prepolymer were stirred and heated to 120 °C until a homogeneous system formed. This homogeneous system was then transferred to a vacuum kneader held at 100 °C and then kneaded for 25 min to obtain a fully degassed epoxy-modified BMI/DABPA prepolymer.

### 2.3. Preparation of Epoxy-Modified and Toughened BMI Structural Adhesive

To prepare epoxy-modified and PEK-C-toughened BMI structural adhesives, PEK-C was weighed according to the formulations listed in Table 1. Specifically, DABPA was heated to 200 °C in a three-necked round bottom flask equipped with an agitator. PEK-C was added and stirred until it melted, and a homogeneous system formed. BMI was added after the temperature was reduced to 135 °C. Then, DGEBA/DDS mixture was added to the mixture after all the BMI had melted, then the well-mixed prepolymer was transferred to a vacuum kneader held at 100 °C and kneaded for 25 min to obtain a fully degassed modified BMI resin. For the preparation of epoxy-modified, PEK-C- and core–shell polymer-toughened BMI structural adhesive, DABPA was heated to 200 °C in a three-necked round bottom flask equipped with an agitator. PEK-C was added and stirred until it melted, and a homogeneous system formed. BMI was added after the temperature was reduced to 135 °C. Then, DGEBA/DDS mixture and core–shell polymer were added to the mixture after all BMI melted, and the well-mixed prepolymer was transferred to a vacuum kneader holding at 100 °C and kneaded under vacuum for 25 min to obtain a fully degassed modified BMI resin. The modified BMI resin was then transferred to a two-roll calender held at 90 °C to prepare the BMI adhesive films with a nylon fabric as the carrier. The thickness of the film adhesive was 0.20 mm.

### 2.4. Preparation of Adhesively Bonded Samples

We prepared the bonded aluminum alloy samples by anodizing the aluminum alloys and rinsing the aluminum honeycombs with acetone before use. The modified BMI adhesive was sandwiched between the substrates with an applied curing pressure of 0.3 MPa. Curing was achieved in an oven with curing conditions of 160 °C/1 h, followed by 180 °C/1 h, and finally at 200 °C/3 h. The co-cured composite samples were prepared according to the modified method reported in our previous work [27]. Only BMI-based carbon fiber prepreg and modified BMI film adhesives were used in the preparation process. The process was conducted in an autoclave under a vacuum with an applied curing pressure of 0.6–0.7 MPa. The curing conditions were the same as those described above.

### 2.5. Characterization

The reaction properties were characterized by differential scanning calorimetry (DSC) on Q20 equipment from TA. The sample was heated from 25 to 350 °C at a heating rate of 10 °C/min. The rheological properties were evaluated using a DHR-1 rheometer with a heating rate of 3 °C/min and a frequency of 1 Hz. The glass transition temperature (T_g_) of the modified resin was determined by dynamic mechanical analysis (DMA) on Q800 equipment (TA Instruments) with heating from 25 to 350 °C at a rate of 5 °C/min and a frequency of 1 Hz. Thermogravimetric analysis (TGA) was performed on Q500 equipment at a heating rate of 10 °C/min from 25 to 800 °C. Scanning electron microscopy (SEM) was performed at an acceleration voltage of 10 kV. Lap-shear tests of the adhesively bonded metal joints were performed on an INSTRON 4045 universal testing machine according to the ASTM D1002-10 test standard. Peel tests were also performed using INSTRON 4467 according to the ISO 4578-1997 standard. Double shear tests of adhesively co-cured composite joints were performed on an INSTRON 4467 universal testing machine according to the ASTM D3528 standard. Atomic force microscopy (AFM) nanomechanical mapping measurements were performed in QNM (quantitative nanomechanics) mode under ambient conditions using Dimension Icon (Bruker).

## 3. Results and Discussion

**Figure 2:**
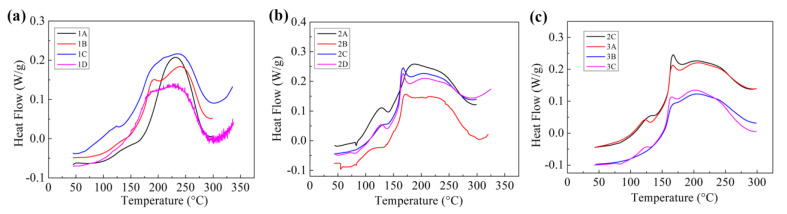
DSC curves of different modified BMI adhesives: (**a**) DSC curves of epoxy-modified BMI resin; (**b**) DSC curves of PEK−C-toughened epoxy-modified BMI resin; and (**c**) DSC curves of PEK−C/core–shell polymer co-toughened epoxy-modified BMI resin.

The reaction characteristics of different modified BMI resins were characterized by non-isothermal DSC, as shown in Figure 2. BMI/DABPA resin had a high reaction temperature with a peak temperature of 230 °C. DGEBA/DDS had little effect on the reaction peak temperature of BMI/DABPA resin, whereas it increased the reaction rate of modified BMI resin at low temperatures, with resulting wide-peak DSC curve characteristics (Figure 2a). This is because DGEBA and DDS can react and release the reaction heat at lower temperatures compared to BMI/DABPA, causing the epoxy-modified system to exhibit significant heat flow at lower temperatures. The heat released by DGEBA and DDS can accelerate the reaction of the modified BMI system, resulting in a lower curing reaction temperature. For PEK-C-toughened epoxy-modified BMI resin and PEK-C/core–shell polymer co-toughened epoxy-modified BMI resin; it was found that the addition of a toughening agent did not change the curing behavior of the modified BMI resin (Figure 2b,c). PEK-C-toughened epoxy-modified BMI resin exhibited an obvious shoulder peek at around 175 °C. This may be due to the fact that PEK increases the viscosity of the resin system, and the reaction heat generated at lower temperatures is difficult to diffuse efficiently, thereby accelerating the reaction between DGEBA and DDS and generating more heat.

**Figure 3:**
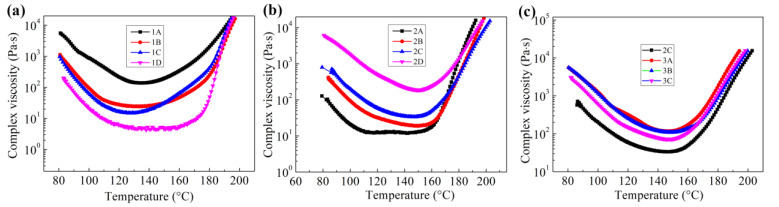
Rheological properties of different modified BMI adhesives: (**a**) Viscosity curves of epoxy-modified BMI resin as a function of temperature; (**b**) Viscosity curves of PEK-C-toughened epoxy-modified BMI resin as a function of temperature; and (**c**) Viscosity curves of PEK-C/core–shell polymer co-toughened epoxy-modified BMI resin as a function of temperature.

One of the main purposes of using epoxy resin to modify BMI resin is to improve properties relevant to the process performance. Figure 3a shows the influence of DGEBA/DDS on the rheological properties of BMI/DABPA. DGEBA/DDS reduces the viscosity of the modified BMI resin and widens the temperature range over which low viscosity is maintained. The minimum viscosity of the modified BMI resin decreases from 138.5 to 4.5 Pa·s when the DGEBA/DDS content is increased from 0 to 78 phr. The viscosity of the modified BMI resin containing 78 phr of DGEBA/DDS was 9.0–20.7 Pa·s in the range of 100–170 °C. Low viscosity characteristics provide the modified BMI with good fluidity and wetness for bonding substrates. In addition, the wider process window can increase the operation time of the modified BMI adhesive in practical applications. As a thermoplastic, PEK-C can be used as both a toughening and film-forming agent, which is very important for preparing structural adhesive films. Figure 3b shows the viscosity changes as a function of PEK-C content at different temperatures. The viscosity of modified BMI resin increases with an increase in PEK-C due to the high melt viscosity of PEK-C. The lowest viscosity of the modified BMI resin increases from 4.5 to 189.2 Pa·s when the PEK-C content is increased from 0 to 60 phr. Although the addition of PEK-C increases the viscosity of the modified BMI resin, which affects the fluidity of the adhesive, it can improve the film-forming properties in manufacturing structural adhesive films. However, when the PEK-C content was 60 phr, the viscosity of modified BMI resin was higher than 1745 Pa·s when the temperature was <100 °C. These conditions are not conducive to manufacturing the film and might reduce its wettability to the substrate. Based on the rheological analysis, the PEK-C dosage in modified BMI resin was 40 phr. To design modified BMI adhesives, we used the core–shell polymer as a secondary toughening agent to improve the adhesive’s toughness without insignificantly increasing its viscosity. Figure 3c shows the influence of the core–shell polymer on the viscosity of the modified BMI. The viscosity of the BMI adhesive showed a slight increase with the increasing content of core–shell polymer.

**Figure 4:**
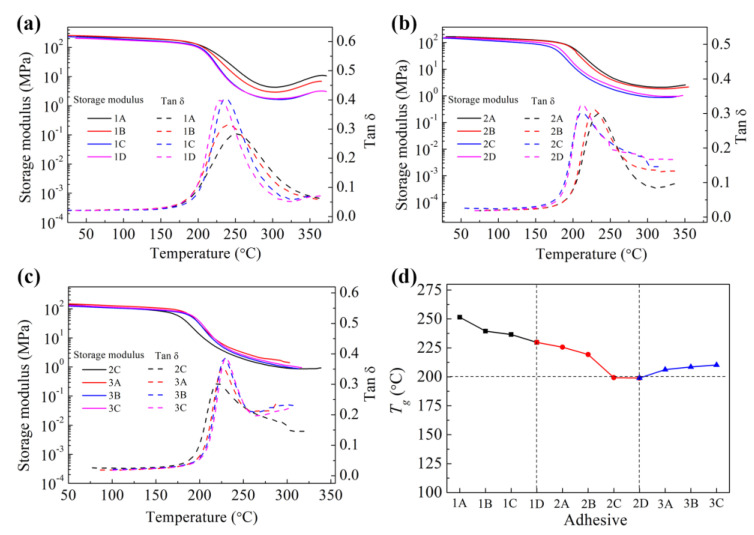
DMA curves of (**a**) epoxy-modified BMI resin; (**b**) PEK−C-toughened epoxy-modified BMI resin; and (**c**) and PEK−C/core–shell polymer co-toughened epoxy-modified BMI resin. (**d**) *T_g_*s of different modified BMI adhesives.

The thermal stability of different modified BMI systems was studied using the DMA method, and the results are shown in Figure 4. The DGEBA/DDS slightly reduced the glass transition temperature of the modified BMI resin from 251.5 to 229.8 °C (Figure 4a) because the heat resistance of the epoxy resin was lower than that of the BMI resin. However, the use of DDS, a high heat resistance curing agent, results in a slight reduction in the glass transition temperature of epoxy-modified BMI resin. Therefore, the modified BMI resin maintains good thermal stability. The storage modulus of cured modified BMI slightly decreased, and the tan delta value increased. As a thermoplastic, there is no crosslinking point between the molecular chains of PEK-C, reducing the glass transition temperature of modified BMI resin from 229.8 to 198.9 °C (Figure 4b). Due to the excellent heat resistance of PEK-C, the glass transition temperature of modified BMI resin can exceed 198 °C. The glass transition temperature of the PEK-C/core–shell polymer co-toughened BMI resin increases slightly after curing because the core–shell polymer contains groups that react with the matrix (Figure 4c).

**Figure 5:**
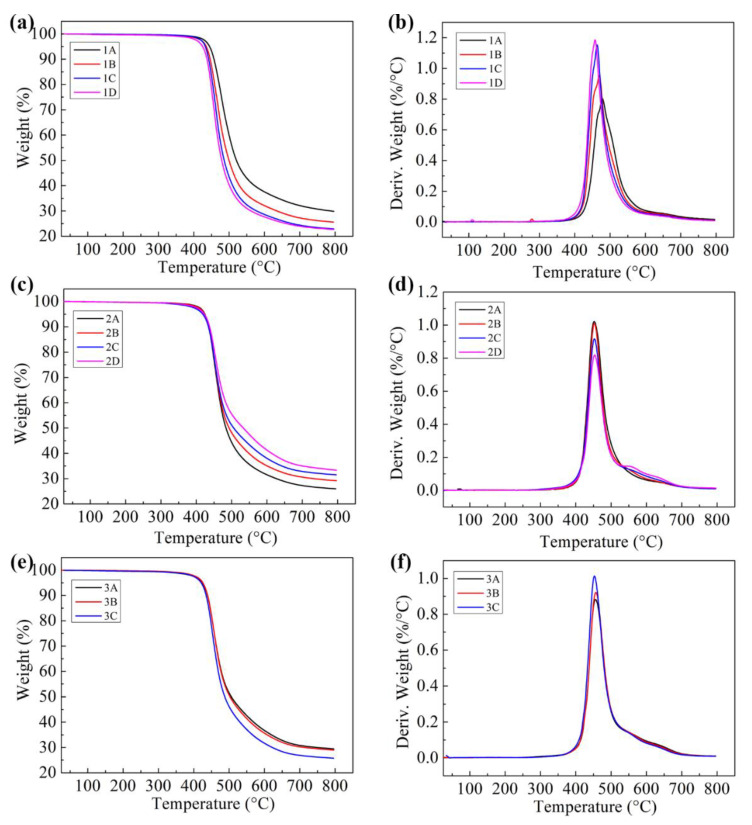
TG and DTG curves of different modified BMI adhesives: (**a**,**b**) epoxy-modified BMI resin; (**c**,**d**) PEK-C-toughened epoxy-modified BMI resin; and (**e**,**f**) PEK-C/core–shell polymer co-toughened epoxy-modified BMI resin.

Figure 5 shows TG and DTG curves of different modified BMI resins in nitrogen. The temperatures at 5% weight loss (T_5%_) and the maximum decomposition rate (T_max_) of BMI/DABPA are 444.7 and 479.5 °C, respectively, demonstrating excellent heat resistance. The maximum decomposition rate is BMI/DABPA 0.8%/°C, which indicates a slow rate of thermal decomposition. The high cross-linking density of BMI resin and its rigid molecular structure account for its excellent heat resistance. The T_5%_ and T_max_ of modified BMI resin decrease after the addition of epoxy resin. When the content of epoxy resin was 78 phr, T_5%_ and T_max_ were 424.9 and 456.2 °C, respectively, and the maximum decomposition rate increased to 1.18%/°C. The heat resistance of epoxy-modified BMI resin was reduced, but T_5%_ is higher than 424 °C, indicating excellent heat resistance (Figure 5a,b). The addition of PEK-C did not reduce T_5%_ of the modified BMI resin and slightly increased its T_max_ and decreased the maximum decomposition rate (Figure 5c,d). Similarly, the addition of core–shell polymers showed less reduction in the heat resistance characteristics of the modified BMI resin (Figure 5e,f), and the data for different modified BMI resin systems are shown in Table 2. The above studies indicate that the modified BMI resin maintains excellent thermal stability.

**Figure 6:**
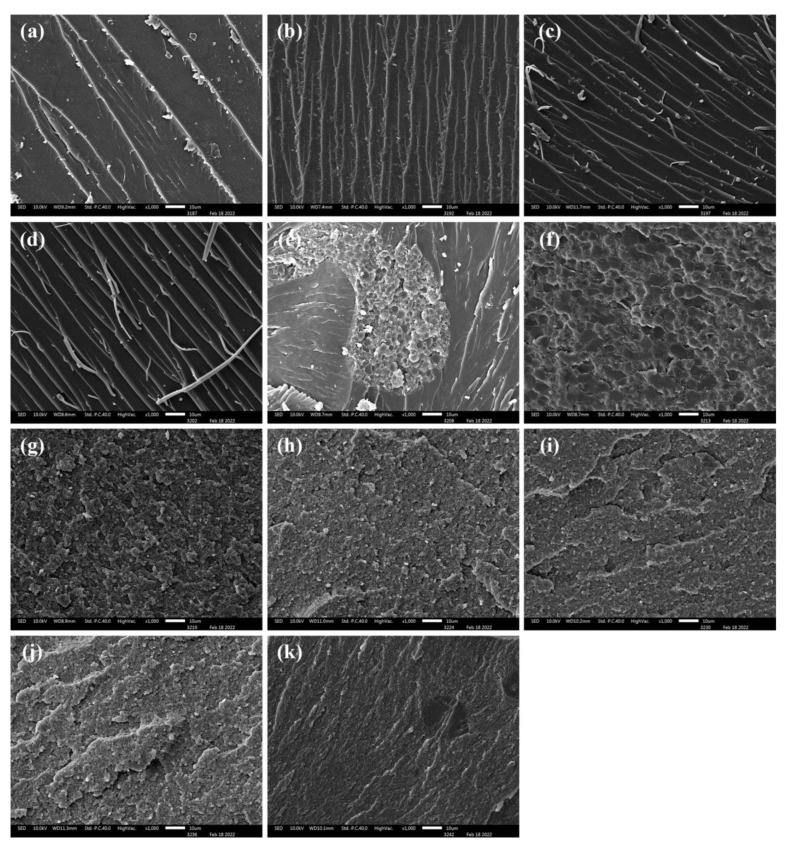
SEM images of different modified BMI adhesive systems: (**a**) 1A system; (**b**) 1B system; (**c**) 1C system; (**d**) 1D system; (**e**) 2A system; (**f**) 2B system; (**g**) 2C system; (**h**) 2D system; (**i**) 3A system; (**j**) 3B system; (**k**) 3C system.

The microtopography of the cured modified BMI’s cross-sections was studied by SEM, as shown in Figure 6. The change in the toughness of the cured resin can be explored by the topography characteristics of the cross-section. Pure BMI resin has a high cross-linking density and low toughness, resulting in brittle fracture characteristics (Figure 6a). The SEM results indicate that the addition of epoxy resin does not significantly increase the toughness of BMI resin due to the high rigidity and cross-linking density of the DGEBA/DDS system (Figure 6b–d). The fracture morphology of the modified BMI resin changes significantly after the addition of PEK-C. The fracture surface of the modified BMI resin exhibits a typical island structure with ductile fracture characteristics when the PEK-C content is 10 (Figure 6e). When the content of PEK-C is increased from 20 to 40 phr, the PEK-C phase is uniformly dispersed in the BMI phase, and the modified BMI resin shows double continuous phase characteristics, resulting in apparent ductile fracture characteristics and an increase in ductile fracture sites (Figure 6f,g). Several small BMI phases become wrapped in PEK-C when PEK-C content reaches 60 phr (Figure 6h), indicating that the modified BMI undergoes phase flipping during curing, which is detrimental to bonding performance. Although high-viscosity thermoplastics can improve the film-forming properties of structural adhesives, they should not be added in excess. Modification of BMI resin with an appropriate amount of core–shell polymer does not significantly affect its phase structure (Figure 6i,j), whereas the addition of an excess of core–shell polymers results in observable flattening in cross-sections (Figure 6k).

**Figure 7:**
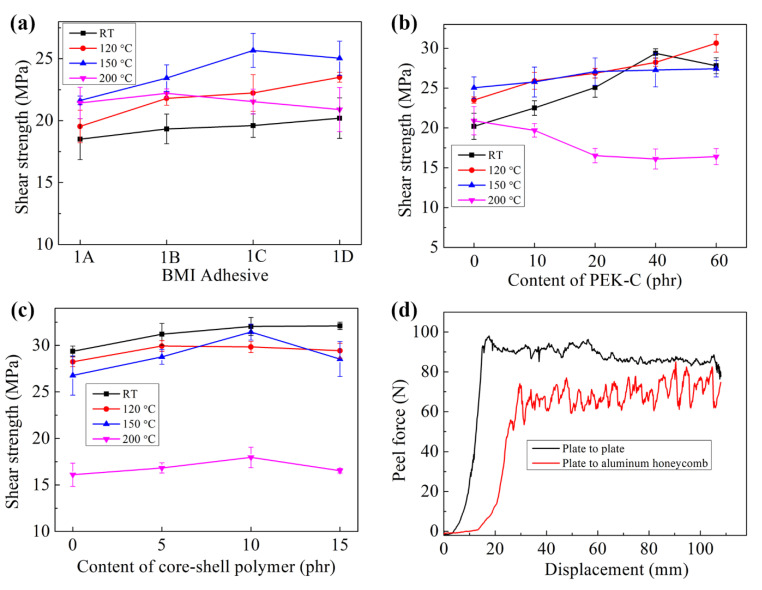
Bonding performance of different modified BMI adhesives. (**a**–**c**) Shear strength of the structural BMI adhesives under different temperatures. (**d**) Peel performance of the optimized structural BMI adhesives (23 °C).

Shear performance is a widely used indicator for evaluating the bonding performance of adhesives. Shear strength can also reflect the toughness of the adhesive. We studied the bonding properties of different modified BMI resin systems based on single-lap shear strength, as shown in Figure 7. The effect of epoxy resin on the shear strength of modified BMI resin is shown in Figure 7a. Modification of BMI resin with epoxy resin resulted in an increase in shear strength at room temperature from 18.5 to 20.2 MPa. Although epoxy resin can improve the bonding performance of modified BMI, the epoxy-modified BMI system is still brittle at room temperature, resulting in a slight increase in shear strength. The shear strength at 120 and 150 °C showed a significant increase with increased epoxy resin content. When the epoxy resin content was 78 phr, the shear strength of the modified BMI system was 23.5 and 25.0 MPa at 120 and 150 °C, respectively. These findings indicate good adhesion performance. Adding epoxy resin slightly decreases the shear strength of the modified BMI resin system at 200 °C, which is attributed to the reduction in the heat resistance of the BMI resin. Figure 7b shows the effect of PEK-C on the bonding performance of modified BMI. PEK-C greatly improves the shear strength of modified BMI at room temperature, 120, and 150 °C. The appropriate amount of core–shell polymer can also improve the shear strength of the modified BMI adhesive without reducing its heat resistance. When the core–shell polymer content is 10 phr, the shear strength of the modified BMI adhesive reaches 32.0 MPa at room temperature and up to 17.9 MPa at 200 °C. These results suggest that the synergistic system, consisting of PEK-C and core–shell polymer, is suitable for producing a high-performing BMI structural adhesive. The peeling performance of an adhesive is another aspect that can be considered to reflect adhesion performance and toughness. We evaluated the peel performance of the optimized BMI adhesive using skin-to-plate and skin-to-core peel tests, as shown in Figure 7d. The optimized adhesive exhibits good peel properties at room temperature with a skin-to-plate peel strength of 4.3 N/mm and skin-to-core peel strength of 3.5 N/mm.

**Figure 8:**
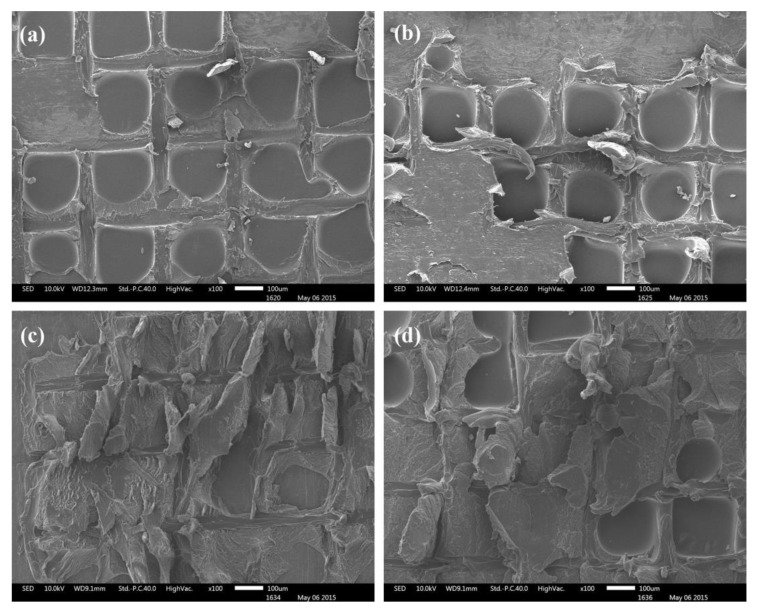
SEM images of BMI adhesive-bonded aluminum joints after testing at different temperatures: (**a**) 23 °C; (**b**) 120 °C; (**c**) 150 °C; and (**d**) 200 °C.

We investigated the failure modes of joints with the modified BMI adhesive by analyzing the interface morphologies formed after failure. Figure 8 shows SEM images of interface morphologies at different test temperatures. The joints show a typical mixture of interface and adhesive cohesion failure at room temperature (23 °C), indicating the excellent cohesive strength and shear properties of the BMI adhesive based on its ability to form a good adhesive interface. As the test temperature rises, the failure modes of joints change significantly. At higher test temperatures, cohesion failure of the adhesive predominates, with significant plastic deformation exhibited. This failure is mainly due to the rigidity of the adhesive decreasing with increasing test temperature. Moreover, plastic deformation tends to occur when the adhesive is subjected to tensile and shear forces. Although the adhesive’s decreased rigidity and cohesive strength at higher temperatures are one of the main reasons for its decreased shear strength, the shear strength of the modified BMI adhesive at 150 °C is comparable to its shear strength at room temperature. Furthermore, the joint did not undergo significant interface damage at high temperatures, indicating that the structural adhesive can form a strong bonding interface with the CFRP material.

**Figure 9:**
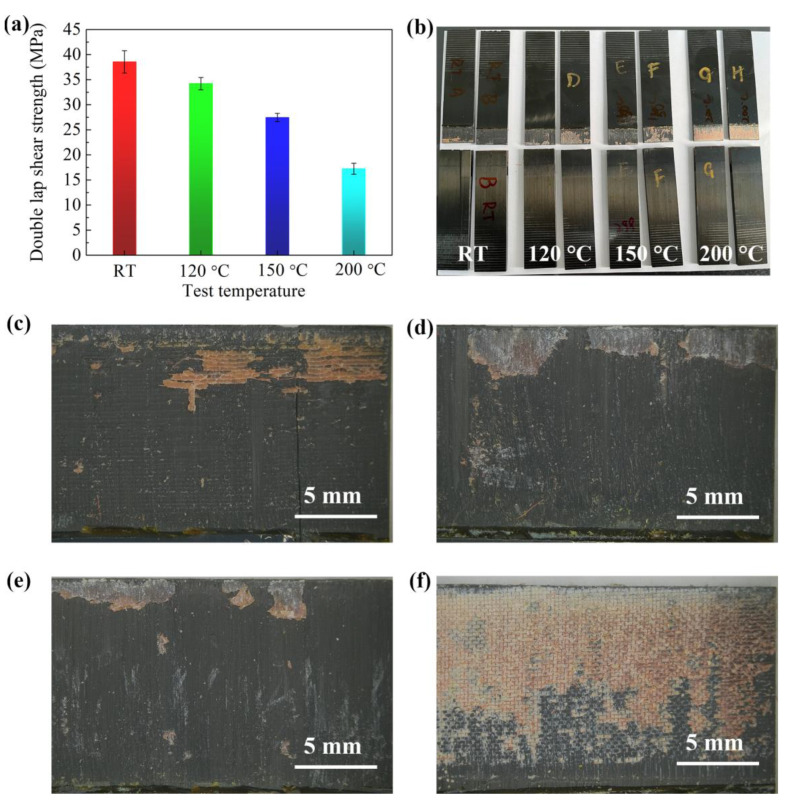
(**a**) Double lap shear strength of composite joints at different test temperatures. (**b**) Photos of composite joints after testing. The failure mode of the co-cured composite joints: (**c**) 23 °C; (**d**) 120 °C; (**e**) 150 °C; and (**f**) 200 °C.

Co-cured BMI composite double lap joints were prepared using the autoclave process. The shear strength of composite joints at different temperatures was tested to evaluate the bonding performance of the modified structural BMI adhesive to the composite, as shown in Figure 9a. Adhesive-bonded composite joints exhibit shear strength of up to 38.6 MPa at room temperature. The double lap shear strength of composite joints decreases as the test temperature increases but is still as high as 17.3 MPa at 200 °C. The above results show that the modified BMI structural adhesive we prepared has excellent properties for bonding BMI CFRP. The failure mode of the adhesively bonded composite joints is also important, as failure sometimes occurs between the composite layers. Adhesive-bonded composite joints exhibit different failure modes under different test temperature conditions (Figure 9b). The composite joints exhibit mixed adhesive and interfacial phase failures at room temperature (23 °C) with a small proportion of composite failure. These findings are mainly due to the rigid and brittle properties of adhesives and composite matrix resins at room temperature. At 120 and 150 °C, a large proportion of composite failure occurs in composite joints, which we attribute to the good interfacial phase formed between the modified BMI adhesive and prepreg during the co-curing process. At 200 °C, composite adhesive joints mostly exhibit adhesive cohesion failure. Therefore, the shear strength of composite joints at 200 °C is comparable to the intrinsic shear strength in tests of aluminum alloy joints. Our results indicate that the prepared modified BMI structural adhesive has comparable performance to BMI composites at a service temperature of around 200 °C. This finding is essential to confirm the high bonding performance of the manufactured joints.

**Figure 10:**
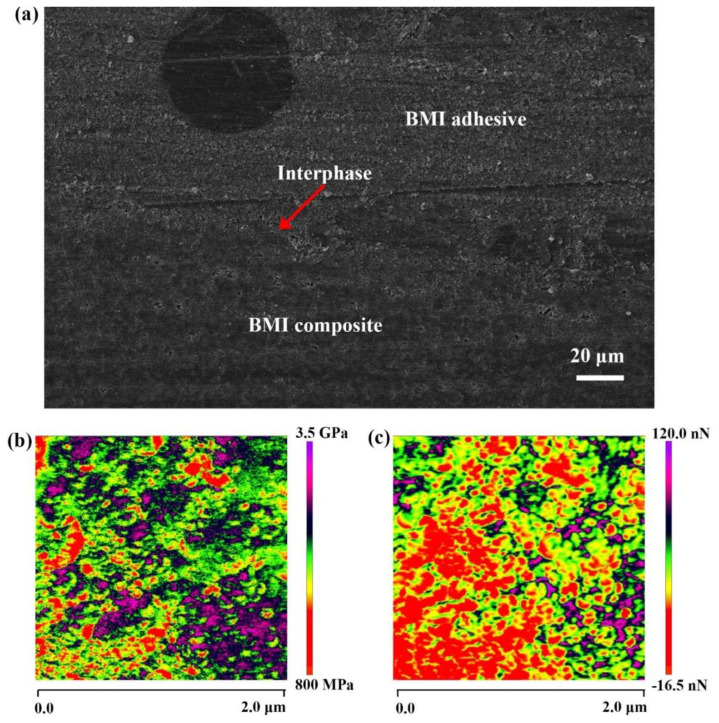
(**a**) SEM image of the bonding interface in the BMI adhesive-bonded composite joint. (**b**) Modulus mapping of the cured BMI adhesive. (**c**) Adhesion to the AFM probe mapping of the cured BMI adhesive.

An SEM image of the bonding interface in the composite joint is shown in Figure 10a, indicating the modified BMI adhesive layer in the composite joint. One of the keys to excellent bonding performance is the thin bonding interface layer, which can be seen between the BMI adhesive and the composite. The bonding interface is an intermediate bridge connecting the adhesive layer to the composite material, mainly through mechanical interlocking and covalent bonding. The strong forces involved in the binding of this bridge result in the formation of mechanically stable and thermodynamically stable composite joints. To better understand the phase structure and interface bonding between the phases of the cured BMI adhesive layer in the composite joint, we performed AFM nanomechanical mapping measurements in QNM mode, as shown in Figure 10b,c. Different modulus distributions represent different phase structures, and our results show that different phase structures exist in the optimized BMI adhesive and are uniformly distributed. At the interface of different phases, adhesion to the AFM probe is gradual and evenly distributed. This gradient phase structure and good interfacial bonding between phase structures are the keys to high-performance modified BMI adhesives.

## 4. Conclusions

In conclusion, we prepared a modified BMI structural adhesive with epoxy-modified BMI as the matrix and PEK-C and core–shell polymers as synergistic toughening agents. DGEBA/DDS epoxy resin improves the process and bonding properties of BMI adhesive but slightly reduces thermal stability. PEK-C and core–shell styrene-butadiene rubber were used as toughening agents in synergistically improving the toughness and bonding performance of the modified BMI adhesive system with the maintenance of heat resistance. The optimized BMI structural film adhesive was obtained using 78 phr of epoxy, 40 phr of PEK-C, and 10 phr of core–shell styrene-butadiene rubber. The optimized BMI adhesive exhibits good thermal stability, improved bonding properties, and a high T_g_ of 208.6 °C and T_5%_ of 425.4 °C. Most importantly, the aluminum joints bonded with optimized BMI adhesive exhibited a satisfactory and high intrinsic shear strength of 32.0 MPa at room temperature and up to 17.9 MPa at 200 °C. The BMI adhesive-bonded composite joint exhibited a high double-lap shear strength of 38.6 and 17.3 MPa at room temperature and 200 °C, respectively, indicating effective bonding and excellent heat resistance. The prepared BMI adhesive has potential applications in bonding high-temperature-resistant BMI CFRP.

## Figures and Tables

**Table 1:** Formula composition of different modified BMI adhesives.

Adhesive	BMI/DABPA (phr)	DGEBA/DDS (phr)	PEK-C (phr)	Core–Shell Polymer (phr)
1A	100	0	0	0
1B	100	26	0	0
1C	100	52	0	0
1D	100	78	0	0
2A	100	78	10	0
2B	100	78	20	0
2C	100	78	40	0
2D	100	78	60	0
3A	100	78	40	5
3B	100	78	40	10
3C	100	78	40	15

**Table 2:** Characteristic heat resistance data of the BMI adhesives.

Adhesive	*T*_g_ (°C)	*T*_d5%_ (N_2_, °C)	*T*_dmax_ (N_2_, °C)	Maximum Degradation Rate (N_2_, %/°C)
1A	251.5	444.7	479.5	0.80
1B	239.5	435.3	467.7	0.95
1C	236.6	432.4	461.3	1.15
1D	229.8	424.9	456.2	1.18
2A	225.6	424.3	451.9	1.02
2B	219.3	423.7	452.6	1.01
2C	199.3	418.4	452.5	0.92
2D	198.9	422.8	453.5	0.82
3A	206.2	424.4	455.8	0.88
3B	208.6	425.4	456.8	0.92
3C	210.2	421.1	452.6	1.01

## Data Availability

Data is available from the author on request.
